# American Medical Association

**Published:** 1851-03

**Authors:** 


### ARTICLE V.

### AMERICAN MEDICAL ASSOCIATION.

The time for the next meeting of this National Medical Assembly is drawing near, and by the time of our next issue of the Journal, Delegates will be wending their way to Charleston, S. C.

We hope our State will be fully represented by delegates from her various medical societies.

There is now no time to be lost in preparation for sending representatives by those bodies that have not already made appointments.

